# Innovative Mycelium Bio-Composites (MB) from Birch Sanding Dust and Chitosan with Enhanced Heavy Metals Sorption Properties

**DOI:** 10.3390/ma19081629

**Published:** 2026-04-18

**Authors:** Oskars Bikovens, Anrijs Verovkins, Ilze Irbe

**Affiliations:** Latvian State Institute of Wood Chemistry, Dzerbenes Street 27, LV-1006 Riga, Latvia; anrijs.verovkins@kki.lv (A.V.); ilze.irbe@kki.lv (I.I.)

**Keywords:** mycelium bio-composites, chitosan, Cu(II) adsorption, Cd(II) adsorption, adsorption isotherms

## Abstract

Chitosan is a well-known heavy metal biosorbent and was incorporated into birch sanding dust mycelium bio-composites (MBs). The chitosan-hybridized MBs with different chitosan contents were characterized by microscopy, porous structure analyses (specific surface area and total pore volume), pH_pzc_, functional group content, and FTIR. Microscopy did not reveal any antifungal effect of chitosan on *Trametes versicolor*. The porous structure of the MBs decreased after hybridization with chitosan. The FTIR spectra and functional group analyses confirmed the presence of chitosan amino groups in the MBs. The chitosan-hybridized MBs were subjected to the adsorption of heavy metals, namely Cu(II) and Cd(II), and the removal percentage and adsorption isotherms were evaluated. Adsorption isotherms were analyzed using the Freundlich and Langmuir models. The results showed a significant increase in the maximum monolayer adsorption capacity for Cu(II), calculated using the Langmuir equation, from <2 mg/g for raw BSD and basic MB without chitosan to 19 mg/g for the MB with 15% chitosan. In the case of Cd(II), no significant increase in adsorption capacity was observed. These findings indicate that hybridization of MBs with chitosan is a promising approach to improve the Cu(II) adsorption capacity of MBs.

## 1. Introduction

Wastewater treatment is one of the global problems, and it is directly related to Sustainable Development Goal 6 (Clean Water and Sanitation). Wastewater treatment has knock-on effects on health (SDG 3), life below water (SDG 14), climate action (SDG 13), and inequality (SDG 10). Heavy metals are assessed as a high-priority contaminant due to their toxicity, persistence, and potential for bioaccumulation. Insufficiently treated industrial wastewater is one of the largest sources of heavy metal contamination in water [1,2]. There are several remediation methods for heavy metal-contaminated wastewater, such as membrane filtration, coagulation, precipitation, and adsorption. Biosorption is a remediation method that coincides with the principles of “green chemistry” or “sustainable chemistry” and has some advantages compared to chemical sorbents. Bacteria, fungi, algae, and different natural and agricultural biomasses have been used for heavy metal removal from wastewaters [2,3].

Chitosan is a linear biopolymer composed of β-(1 → 4) linked D-glucosamine and N-acetyl-D-glucosamine that is obtained from one of the most abundant natural biopolymers, chitin, by deacetylation. Chitosan has various applications in different areas, including the food industry, agriculture, medicine, cosmetics, and wastewater purification [4]. Chitosan exhibits good adsorption properties against heavy metal ions, phosphate, dyes, pesticides, etc. [4,5]. Chitosan is a low-cost biosorbent with a high heavy metal adsorption capacity. However, its primary limitations include low mechanical resistance, solubility in acidic solutions, and a nonporous structure [6,7]. To overcome this, different modifications of chitosan have been proposed, including preparation of porous gel beads [8]; synthesis of chitosan nanoparticles; crosslinking of chitosan amino groups with glutaraldehyde, citric acid, or other crosslinking agents [4,6,9]; or crosslinking of chitosan hydroxyl groups with epichlorohydrin [7]. Chitosan properties can be improved by its incorporation into composites. Chitosan composites with activated carbon and polyurethane/chitosan composites have been prepared for removing dye [4]. Kaur incorporated chitosan and carboxymethyl cellulose into graphene oxide nanosheets [10].

Our approach involves the incorporation of a bio-sorbent (chitosan) into growing mycelium to hybridize mycelium bio-composites. Mycelium bio-composites are a relatively new type of material that finds various applications in different areas, including building materials, packaging, insulation, etc. [11,12]. The integration of chitosan into mycelium-based bio-composites offers a sustainable and eco-friendly alternative to the chemical modification of chitosan-based sorbents. The aim of the present study was to obtain a new chitosan/mycelium bio-composite and use it for Cu(II) and Cd(II) adsorption from aqueous solution. Copper is one of the most common heavy metals. Although copper is an essential trace element, it is toxic to humans if the dose is high enough. In the case of cadmium, only negative effects have been found so far [13]. To the best of our knowledge, no study has been conducted on the heavy metal adsorption properties of mycelium bio-composite and chitosan/mycelium bio-composite. The chitosan/mycelium bio-composite was characterized using various physical, physicochemical, and chemical methods. Heavy metal adsorption was studied using the batch method. We hypothesized that the incorporation of chitosan would contribute to increasing the metal uptake by mycelium bio-composites (MBs).

## 2. Materials and Methods

### 2.1. Development of MBs with Chitosan

The birch (*Betula pendula*) sanding dust (BSD) as lignocellulose substrates for the development of MBs was obtained from the plywood producer JSC “Latvijas Finieris” (Latvia). MBs were produced from *Trametes versicolor* mycelium grown on BSD by the modified method [14]. Commercial chitosan from crustaceans, with a deacetylation degree > 90% and medium molecular weight from Jiangsu Aoxin Biotechnology Co., Ltd. (Lianyungang, China) were used for modification of the MBs.

### 2.2. MBs Characterization Methods

The micromorphology of the cross-sections of MB specimens (30 × 30 × 30 mm^3^) was examined using a Leica S9i digital stereo microscope equipped with a 10 MP integrated camera (Leica Microsystems, Wetzlar, Germany) at 50-time magnification. Image acquisition and analysis were performed using Leica LAS V 4.12.0 software, and all images were stored in a PC-based database.

For scanning electron microscopy (SEM) analysis, 2 × 2 × 2 mm^3^ cubes were cut from the specimen cross-sections to evaluate fungal distribution within the internal structure. Chitosan crystals and raw BSD fibers were analyzed as reference materials. Prior to imaging, all samples were sputter-coated with gold using a K550X sputter coater (Emitech, Chelmsford, UK). SEM observations were carried out using a TESCAN VEGA TX microscope (Tescan, Brno, Czech Republic) at 1000× magnification, with samples mounted on graphite tape.

The porous structure was determined for basic MB and chitosan-hybridized MB samples. The porous structure (specific surface area and total volume of micro- and mesopores) of the MBs blends was determined from isotherms of low-temperature nitrogen adsorption-desorption at 77 K (−196.15 °C) on a Nova 4200e device (Quantachrome instruments, Boynton Beach, FL, USA). Specific surface area (S) and volume (V) were calculated by the Brunaer–Emmet–Teller (BET) method.

The points of zero charge (pH_PZC_ values) of the MBs were determined by the pH drift method [15]. The pH was adjusted to a value between 3 and 9 using diluted HCl and NaOH. The initial and final pH (equilibrated for 24 h) were measured by using a FiveEasy pH meter (Mettler Toledo, Schwerenbach, Switzerland). Phenolic, amino, and carboxyl groups were detected in chitosan-hybridized MB samples by conductometric titration. Approximately 40–50 mg of the completely dried sample was weighed and placed into a tightly sealed container. A total of 5.0 mL of 0.1 M NaOH was added. The container was purged with nitrogen, sealed, and the solution was thoroughly mixed. The mixture was left to stand for 24 h. Titration was performed using 0.1 M HCl. The process was carried out automatically using an electrochemical conductometric method, on a CDM210 conductivity meter device with autoburette ABU 901 in complect (Radiometer Analytical SAS, Villeurbanne, France) [16].

Fourier transform infrared (FTIR) spectra of mycelium bio-composites were recorded in KBr (IR grade, Sigma Aldrich, Darmstadt, Germany) pellets by a Nicolet iS50 spectrometer (Thermo Fisher Scientific, Waltham, MA, USA) in the range of 4000 to 450 cm^−1^ with a 4 cm^−1^ spectral resolution and 32 scans. The pellet contained approximately 2 mg of the ball mill MM200 (Retsch, Haan, Germany) ground wood sample and 200 mg KBr. All spectra were normalized to the highest absorption maxima.

### 2.3. Heavy Metal Adsorption by MBs

Copper(II) nitrate trihydrate (p.a.), cadmium nitrate tetrahydrate (p.a.), and chromium(III) potassium sulfate dodecahydrate (p.a.) used in the adsorption experiments were supplied by Lach-Ner (Neratovice, Czech Republic). An ICP multi-element standard solution IV certified reference material was supplied by Merck KGaA (Darmstadt, Germany) and used for the determination of heavy metal concentrations in solutions by atomic absorption spectrometry (AAS).

A Shimadzu AA-6300 (Shimadzu Corporation, Kyoto, Japan) atomic absorption spectrometer equipped with corresponding hollow cathode lamps (Cathodeon, Cambridge, UK) was used to determine Cu(II) and Cd(II) ion concentrations. The wavelengths for the determination of heavy metals were as follows: Cu—324.8 nm and Cd—228.8 nm. The flame type was air-acetylene, with an acetylene flow rate of 1.8 L min^−1^ and an air flow rate of 15 L min^−1^. The instrumental parameters recommended by the manufacturer were used. AAS results were processed with WizAArd version 3.0 (Shimadzu Corporation, Kyoto, Japan) software.

Removal experiments were carried out to screen the efficiency of the MBs as biosorbents. Batch adsorption studies were performed by mixing a fixed mass of biosorbent (0.5 g) with 50 mL of aqueous metal solution (metal concentration 50 mg/L) in polypropylene centrifuge tubes for 24 h at 22 °C under continuous stirring (Sky Line Shaker, ELMI Ltd., Riga, Latvia). The initial pH was adjusted using dilute HNO_3_ or NaOH with a pH meter FiveEasy (Mettler Toledo, Schwerenbach, Switzerland). After sorption, the samples were centrifuged and filtered through a 0.45 μm nylon syringe filter, and the heavy metal concentrations were measured by AAS.

Removal of heavy metals was calculated through Equation (1):

(1) $$Removal\;\%=\frac{C_{o}-C_{e}}{C_{o}}\;\times \;100\%$$

where *C_o_*—the initial concentration of heavy metals (mg/L), and *C_e_*—the concentration of heavy metals at equilibrium (mg/L).

Kinetic studies were carried out with sorption times ranging from 10 min to 24 h.

In the pH studies, the initial pH of the metal solutions was in the range of 5–7. During sorption, the pH was re-adjusted several times until a constant value was achieved using dilute HNO_3_ or NaOH.

The adsorption isotherm model was used to evaluate the performance of the biosorbents. Equilibrium isotherm experiments were carried out at room temperature (22 ± 1 °C). The initial heavy metal concentration was varied from 10 mg/L to 160 mg/L. The sorption time was 24 h with continuous stirring. After this time, the solutions were filtered and analyzed as described above. The equilibrium adsorption capacity of heavy metals, *q_e_* (mg g^−1^), was calculated using Equation (2):

(2) $$q_{e}=\frac{V*(C_{o}-C_{e})}{m}$$

where *V* is the volume of the solution (L), and *m* is the mass of biosorbent (g).

The sorption isotherms were constructed from the *q_e_* and *C_e_* data and described as the Freundlich model (3) and the Langmuir model (4). The Freundlich and Langmuir models are among the most frequently used adsorption models [17]. The Freundlich model is an empirical equation without physical meaning that describes the relationship between the concentration of sorbate in the liquid phase and the surface area of the sorbent. However, as later studies have shown, the Freundlich model describes both chemical adsorption (up to 50% coverage) and physical adsorption well. The Langmuir model describes monolayer adsorption with a homogeneous arrangement of adsorption centers and constant adsorption energy. Despite a number of assumptions, the model allows for the determination of the monolayer adsorption capacity [17]:

(3) $$q_{e}=K_{F}*{C_{e}}^{1/n}$$

(4) $$q_{e}=\frac{q_{m}*K_{L}*C_{e}}{1+K_{L}*C_{e}}$$

where *K_F_* and *n* are Freundlich constants, *q_m_*—the maximum monolayer adsorption capacity estimated by the Langmuir model (mg g^−1^), and *K_L_*—the ratio of the adsorption rate and desorption rate (L × mg^−1^).

The logarithmic forms of the Freundlich Equation (5) and the linear form of the Langmuir Equation (6) were used for the calculation of corresponding constants:

(5) $$\ln q_{e}=\ln K_{F}+\frac{1}{n}*\ln C_{e}$$

(6) $$\frac{C_{e}}{q_{e}}=\frac{C_{e}}{q_{m}}+\frac{1}{K_{L}q_{m}}.$$

The Freundlich model was solved by plotting ln *q_e_* versus ln *C_e_*. The Langmuir equation was solved by plotting *C_e_*/*q_e_* versus *C_e_* [17].

All experiments were performed in triplicate, and the mean value was used.

## 3. Results and Discussion

The obtained basic MB and chitosan-hybridized MB were characterized using microscopy, nitrogen adsorption-desorption isotherms, FTIR spectra, pH_pzc_, and functional group analyses to estimate the effect of chitosan hybridization on the MB surface structure and chemical properties that could be important for heavy metal adsorption.

### 3.1. Characterization of MBs

#### 3.1.1. Microscopy of MBs

**Figure 1:**
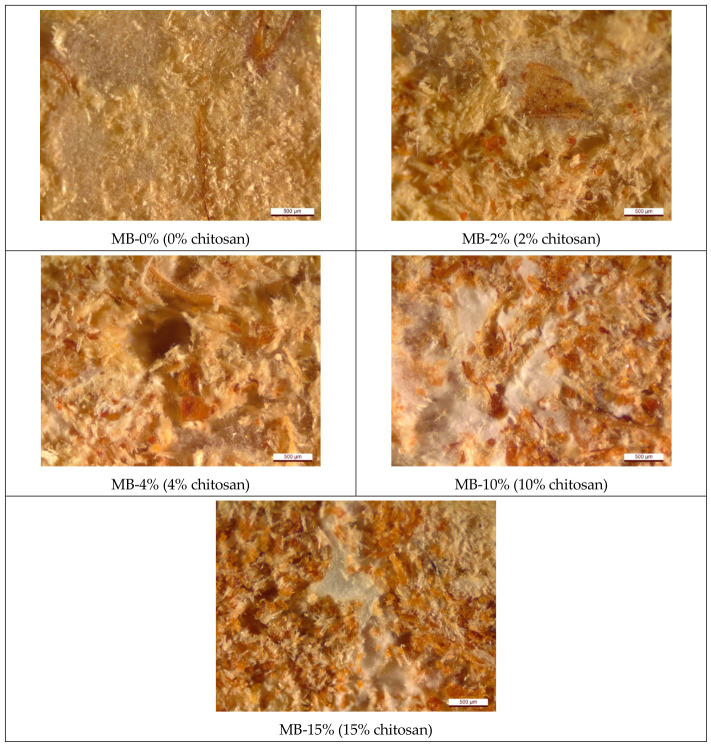
Microstructure of MBs derived from *Trametes versicolor* mycelium and BSD substrate, with chitosan additive at increasing concentrations. Brown chitosan crystals are shown within the structure of MBs. The scale bar shows 500 μm.

Stereomicroscopy and SEM were used to observe chitosan deposition and its interaction with fungal hyphae in the hybridized MB. The results showed that brown chitosan crystals were located within the structure of MB, and fungal growth around them was not restricted (Figure 1).

**Figure 2:**
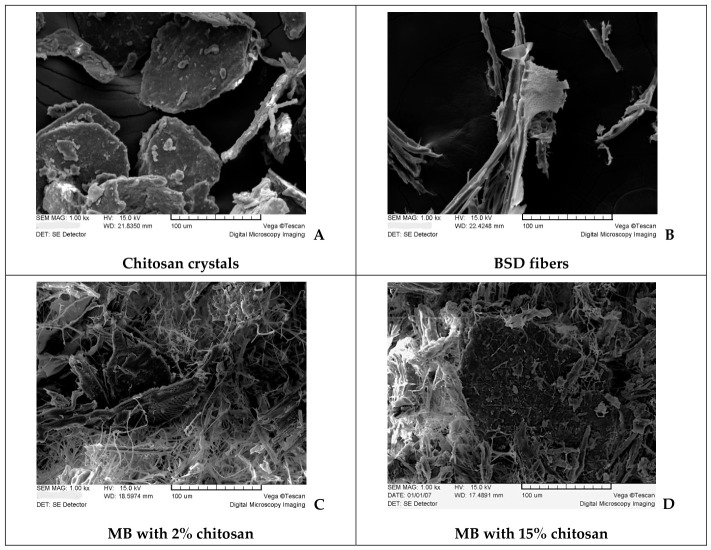
SEM image of chitosan crystals (**A**), BSD fibers (**B**), MB with BSD substrate and chitosan additive at increasing concentrations (**C**,**D**). The scale bar shows 100 μm.

SEM images provided a detailed view of chitosan and BSD microstructure before (Figure 2A,B) and after (Figure 2C,D) MB hybridization with chitosan bio-polymer. The images revealed hyphal distribution both near and over the chitosan crystals.

Despite numerous reports about chitosan’s significant and well-documented antifungal activity against (plant pathogenic) fungi [18], the results obtained did not reveal any significant antifungal effect of chitosan on *Trametes versicolor*.

#### 3.1.2. Porous Structure of MB

The porous structure parameters were calculated (Table 1) from the nitrogen adsorption-desorption isotherm data in our previous study [19]. All studied basic MB samples had a low specific surface area ranging from 2 to 3.3 m^2^·g^−1^, which is higher than that of raw BSD (1.4 m^2^·g^−1^). The obtained results are close to published results for wood, e.g., 2.77 m^2^·g^−1^ for acacia wood [20]. However, the results of water vapor sorption on biomass showed a specific surface area of up to 333 m^2^·g^−1^ for alder wood [21]. The difference between nitrogen and water vapor sorption can be explained by biomass swelling in water vapor.

The specific surface area and pore volume of basic MB (MB-0%) decreased after hybridization with chitosan.

#### 3.1.3. FTIR Spectroscopy

**Figure 3:**
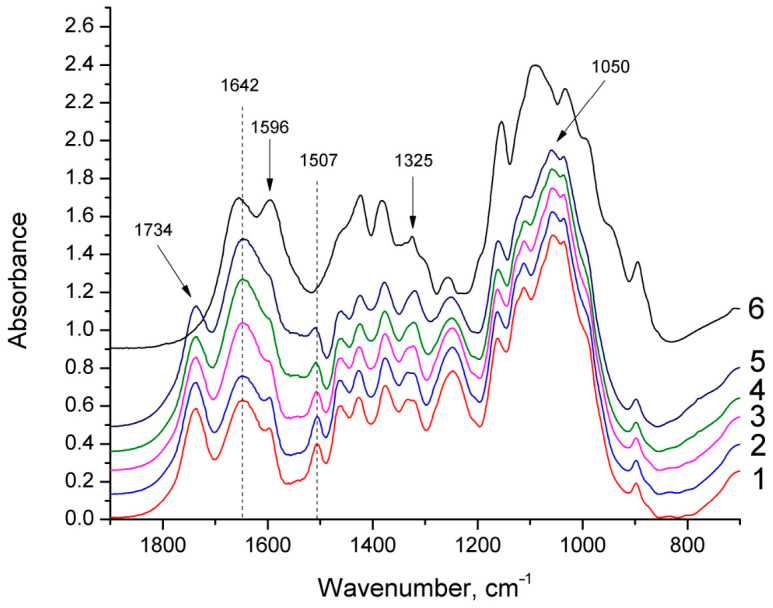
FTIR spectra of basic mycelium bio-composites (MB) and chitosan-hybridized MB (1—basic MB, 2—chitosan content 2%, 3—chitosan content 4%, 4—chitosan content 10%, 5—chitosan content 15%, and 6—commercial chitosan).

The absorption bands of the FTIR spectra used for analysis were assigned in accordance with references [22,23]. The FTIR spectra of the basic MB and chitosan-hybridized MB (Figure 3) showed typical spectra of hardwood and were similar to previously reported FTIR spectra, with a broad peak at 3420 cm^−1^ for OH groups, a region around 3000–2800 cm^−1^ for aliphatic stretching (symmetric and asymmetric stretching of –CH_2_– and –CH_3_ groups), a peak at 1734 cm^−1^ for unconjugated C=O of aliphatic ester groups (mainly acetyl groups), peaks at 1596, 1507, and 1424 cm^−1^ corresponding to aromatic skeletal vibrations of lignin, a peak at 1463 cm^−1^ for asymmetric C–H deformation in –CH_3_ typical of lignin methoxyl groups, and a peak at 832 cm^−1^ for C–H out-of-plane vibrations at the two and six positions of the lignin syringyl ring. The highest absorption maxima in the fingerprint region of both spectra corresponded to C–O, C–O–C, and C–O–H vibrations around 1050 cm^−1^, typical of sugars.

Unlike wood spectra, chitosan FTIR spectra exhibit a strong absorption around 1645 cm^−1^ (C=O stretching of amide I of residual N-acetyl groups) and at 1550 cm^−1^, which corresponds to N–H bending of amide II, as well as a weaker absorption band at 1325 cm^−1^ (C–N stretching of amide III) [24]. With increasing chitosan content in the mycelium composites, absorption also increased in the broad range from 1700 to 1500 cm^−1^, confirming an elevated presence of N-containing compounds (chitosan and/or proteins) in the mycelium composites. A positive correlation was observed between chitosan content in the chitosan-hybridized MB and absorbance at 1642 cm^−1^ (Pearson correlation coefficient ρ = 0.97).

#### 3.1.4. The Point of Zero Charge and Functional Group Analyses

The pH of the point of zero charge (pH_pzc_) for the MBs was between 4.37 and 4.57, which is lower compared to that of raw BSD, as shown in Table 2. The point of zero charge indicates that the MBs have a negative charge above pH_pzc_ (>4.6). The pH_pzc_ value increased after the incorporation of chitosan into the mycelium bio-composites.

The pH of the point of zero charge (pH_pzc_) for the MBs was between 4.37 and 4.57, and it is lower compared with raw BSD, as shown in Table 2. The point of zero charge showed that the MBs have a negative charge beyond pH_pzc_ (>4.6). The pH_pzc_ value increased after incorporation of chitosan into mycelium bio-composites.

Conductometric titration was carried out to determine the mass fraction of functional groups of the MBs (Table 2). The total amount of functional groups in the MBs is higher compared to that of raw BSD. The amount of phenolic OH groups decreased, while carboxylic groups (-COOH) significantly increased in the MBs. There was a trend of increasing amino group (-NH_2_) mass content with the addition of chitosan. The content of phenolic -OH in the chitosan-hybridized MB was lower compared to that of the basic MB. The content of carboxylic groups in the MBs did not show any significant trend of change with varying chitosan percentages.

### 3.2. Heavy Metal Adsorption

#### 3.2.1. Effect of Sorption Time

The effect of the contact time of sorbate and sorbent is one of the most important parameters of sorption experiments. To determine the time required to reach sorption equilibrium, a sorption kinetics experiment was performed over a period of 24 h.

**Figure 4:**
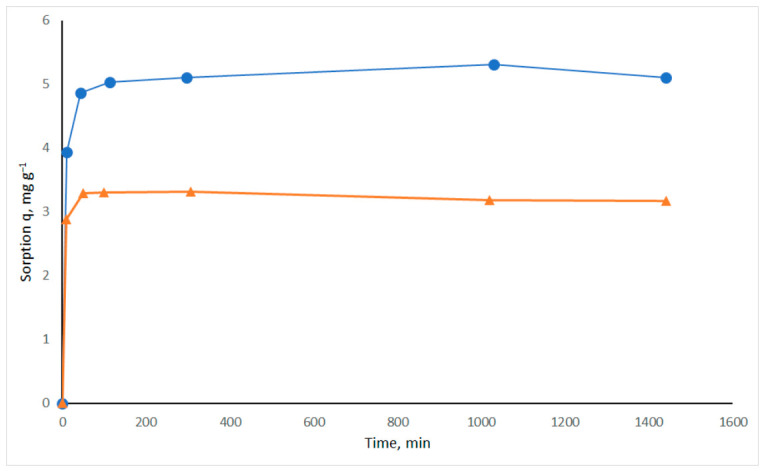
Effect of sorption time on sorption of Cu(II) (blue line) and Cd(II) (orange line) by mycelium bio-composite MB-10% (*m* = 0.5 g; *V* = 50 mL of 50 mg/L Cu(II) or Cd(II); initial pH 5.5 for Cu(II) and 6.0 for Cd(II); *T* = 20 °C).

The effect of contact time between chitosan/MBs and Cu(II) and Cd(II) solutions is shown in Figure 4. Sorption occurs rapidly within the first 10 min. In the case of Cd(II), approximately 87% was sorbed within the first 10 min, while in the case of Cu(II), only approximately 74% was achieved. After 1 h, Cd(II) sorption reaches its maximum value and does not change further, whereas Cu(II) sorption reaches its maximum after 5 h. This is consistent with the two-stage sorption model described by Liu [25]: first, rapid sorption of heavy metals onto the surface occurs, followed by slower diffusion of heavy metal ions into the inner layers. Further experiments were performed over a period of 22–24 h.

#### 3.2.2. Effect of pH

The pH value strongly influences the sorption of heavy metal ions. The effect of pH on the sorption of Cu(II) and Cd(II) onto MBs was determined in neutral and weakly acidic media because, in alkaline media, insoluble hydroxides can form. Cu(II) hydroxide formation occurs already at pH 7 [26], while Cd(II) hydroxide formation occurs at pH > 8 [27].

**Figure 5:**
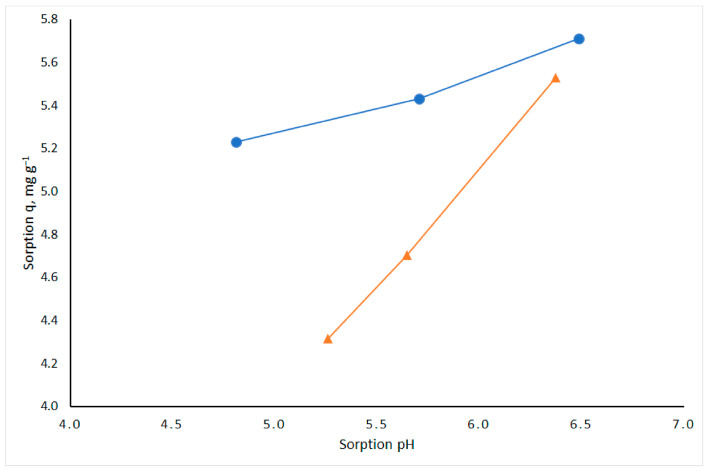
Effect of pH on adsorption of Cu(II) (blue line) and Cd(II) (orange line) by mycelium bio-composite MB-10% (*m* = 0.5 g; *V* = 50 mL of 50 mg/L Cu(II) or Cd(II); *t* = 24 h; *T* = 22 °C).

As the pH of the solution increased from 4.8 to 6.5, Cu(II) sorption increased by 9%, whereas Cd(II) sorption increased by 28% when the pH increased from 5.3 to 6.4 (Figure 5). The sorption experiments were performed at pH values greater than pH_pzc_, where the sorbent surface was negatively charged. These results indicate that Cd(II) sorption is more sensitive to changes in solution pH than Cu(II) sorption.

#### 3.2.3. Removal Experiments

Heavy metal removal experiments were conducted to estimate the impact of the amount of chitosan incorporated into MBs on their sorption properties. The pH values before adsorption were 5.5 and 6.0 for Cu(II) and Cd(II), respectively. After adsorption, the pH decreased to 4.4–4.6 for the Cu(II) solution and to 4.7–4.9 for the Cd(II) solution, which clearly indicates ion exchange between the sorbent and sorbate.

**Figure 6:**
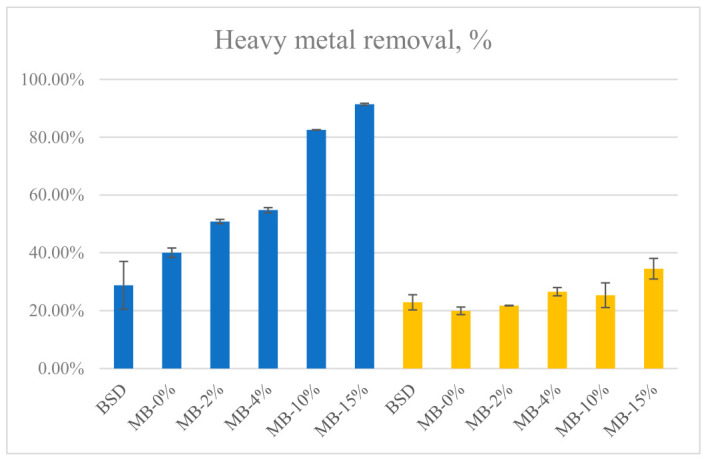
Cu(II) (blue columns) and Cd(II) (yellow columns) removal by raw birch sanding dust (BSD), basic mycelium bio-composites (MB) and chitosan-hybridized MB (% of chitosan content) (*m* = 0.5 g; *V* = 50 mL of 50 mg/L Cu(II) or Cd(II); initial pH 5.5 for Cu(II) and 6.0 for Cd(II); *t* = 24 h, *T* = 22 °C).

Chitosan showed a very high removal capacity for heavy metals compared with MBs under the experimental conditions, 99.8% and 98.6% removal of Cu(II) and Cd(II), respectively. The lowest Cu(II) and Cd(II) removal capacity was shown by raw birch sanding dust (Figure 6). Hybridization of mycelium bio-composites with chitosan significantly increased Cu(II) removal, whereas in the case of Cd(II) ions, an effect was observed only at a high loading of chitosan. Under the experimental conditions used, the highest equilibrium sorption capacity was 4.7 mg g^−1^ for Cu(II) and 1.3 mg g^−1^ for Cd(II) for the MB with 15% chitosan content.

#### 3.2.4. Adsorption Isotherms

Metal adsorption onto the bio-composites significantly increased with an increase in the initial metal concentration until reaching a sorption plateau. The adsorption isotherms were described by the Freundlich or Langmuir model. However, at higher metal concentrations (more than 100–140 mg/L), deviations from I-type isotherms were observed. In the case of Cu(II) and Cd(II), the metal sorption capacity increased significantly. The Langmuir model is useful for describing monolayer adsorption, whereas the Freundlich model describes physical adsorption and chemical adsorption with an equilibrium coverage fraction of about 50% [17]. At high metal concentrations, these adsorption models do not fit the experimental data. The maximum sorption capacity for Cu(II) was 15.9 mg/g on MB-15%, and for Cd(II) it was 5 mg/g on MB-15%.

**Figure 7:**
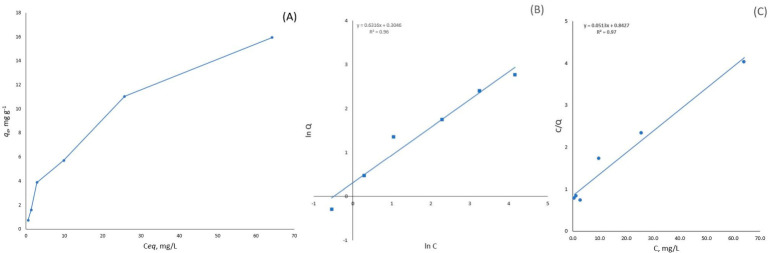
Adsorption isotherm of Cu(II) on chitosan hybridized mycelium bio-composite MB-15% (*m* = 0.5 g; *V* = 50 mL; initial pH 5.5; *t* = 24 h; *T* = 22 °C). (**A**) Experimental adsorption isotherm, linear form of the Freundlich (**B**) and Langmuir (**C**) isotherms.

**Figure 8:**
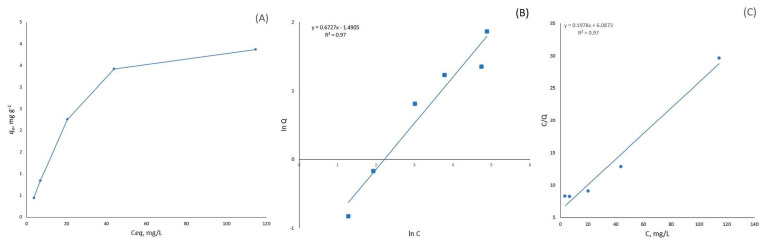
Adsorption isotherm of Cd(II) on chitosan hybridized mycelium bio-composite MB-15% (*m* = 0.5 g; *V* = 50 mL; initial pH 6.0; *t* = 24 h; *T* = 22 °C). (**A**) Experimental adsorption isotherm, linear form of the Freundlich (**B**) and Langmuir (**C**) isotherms.

Figure 7 and Figure 8 and Table 3 show that the Cu(II) adsorption capacity on mycelium bio-composites was directly dependent on the chitosan content in the MBs. On the other hand, no relation was observed between the chitosan content in the mycelium bio-composites and Cd(II) adsorption. This could be explained by the reaction between the chitosan amino groups and Cu(II) ions. Cu(II) ion adsorption by pure chitosan showed a very high sorption capacity of >20 mg g^−1^, and the removal of Cu(II) reached 99%. In contrast, the stability of Cd(II) complexes was much lower compared to that of Cu(II)-amino group complexes.

The Freundlich model proposes multilayer sorption with heterogeneous energetic and surface distribution. The Freundlich isotherm parameters are presented in Table 3. The 1/n parameter is related to the intensity of sorption. The 1/n values are in the range of 0 < 1/n < 1, which indicates a heterogeneous surface structure with an exponential distribution of active sites [28]. The highest relative adsorption capacity (K_F_) for Cu(II) and Cd(II) adsorption was observed for MB 15%. The Freundlich sorption model does not describe all the obtained sorption isotherms with the same accuracy, as the coefficient of determination varied over a wide range (from 0.99 to 0.66) for the linear Equation (5).

The Langmuir sorption model fits the metal sorption data for the mycelium bio-composites better than the Freundlich model. The Langmuir model allows calculation of the maximum monolayer adsorption capacity of the sorbents (q_m_). The highest calculated q_m_ value was approximately 19.5 mg g^−1^ for Cu(II) onto the MB-15% biosorbent. In the case of Cd(II), no significant increase in the maximum monolayer adsorption capacity was observed. Cu(II) and Cd(II) have different hard-soft acid-base characters, which are important for their chelation properties and adsorption. The presence of chitosan amino groups in the MBs enhances Cu(II) adsorption but does not significantly affect Cd(II) adsorption under experimental conditions (at pH ~5). This could be a result of the different chemical nature, specifically the Hard and Soft Lewis Acid-Base (HSAB) properties of Cu(II) and Cd(II) ions, and their interaction with MBs and chitosan functional groups. Cu(II) is classified as a borderline Lewis acid and binds strongly to borderline and strong bases, such as the amino groups of chitosan, unlike Cd(II), which is a soft Lewis acid and has a strong affinity for soft bases [29].

**Figure 9:**
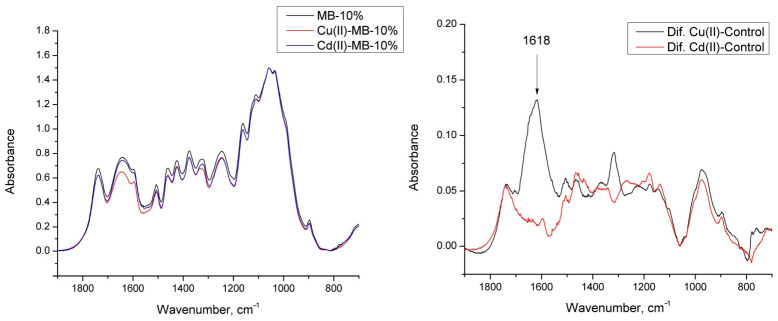
FTIR spectra of chitosan/mycelium bio-composite (MB-10%) before and after Cu(II) (Cu(II)-MB-10%) or Cd(II) (Cd(II)-MB-10%) sorption (**left side**) and their differential spectra ((**right side**), Dif. Cu(II)-Control and Dif. Cd(II)-Control, corresponding)).

This assumption was confirmed by the FTIR spectra of the chitosan/mycelium bio-composite before and after exposure to Cu(II) or Cd(II). Comparing the FTIR spectra before and after sorption (Figure 9, left side), it can be seen that in both cases there is a decrease in absorption in the carboxyl group region (approximately 1734 cm^−1^). After sorption of Cu(II) ions onto the chitosan/mycelium bio-composite, the FTIR spectrum additionally shows a significant decrease in absorption in the range from 1650 to 1590 cm^−1^, which corresponds to the deformation vibrations of primary amines [22]. The differential FTIR spectra (Figure 9, right side) clearly show the difference between the Cu(II) and Cd(II) sorption mechanisms on the chitosan/mycelium bio-composite, as well as the interaction of Cu(II) ions with chitosan amino groups, which was not observed for Cd(II) ions under experimental conditions (at pH ~5).

## 4. Conclusions

This study presents a new approach for biosorbent development using mycelium bio-composites hybridized with chitosan. The obtained mycelium bio-composites containing chitosan possess amino groups, which are important for heavy metal sorption. Cu(II) sorption capacity increased more than tenfold following the incorporation of chitosan into MBs, reaching a maximum biosorption capacity of 19.5 mg g^−1^. The effect of chitosan incorporation on Cd(II) biosorption was less clear. This can be explained by the different Hard and Soft Lewis Acid-Base (HSAB) properties of Cu(II) and Cd(II) and their different preferred chelating agents based on HSAB theory, as confirmed by FTIR spectra.

## Figures and Tables

**Table 1:** Specific surface area (S_BET_) and total volume (V) of pores of birch sanding dust (BSD) and mycelium bio-composites (MB) [19].

MB Samples with Chitosan Concentration, %	$S_{\mathrm{BET}},\;m^{2}*g^{-1}$	$V,\;{cm}^{3}*g^{-1}$
BSD	1.4 ± 0.1	3.1 ± 0.1 × 10^−3^
MB-0%	3.3 ± 0.4	5.0 ± 0.3 × 10^−3^
MB-2%	2.8 ± 0.5	4.1 ± 0.5 × 10^−3^
MB-4%	2.7 ± 0.1	4.0 ± 0.1 × 10^−3^
MB-10%	2.6 ± 0.2	3.9 ± 0.2 × 10^−3^
MB-15%	2.0 ± 0.1	3.8 ± 0.1 × 10^−3^

**Table 2:** pH_pzc_ and mass fractions of functional groups in MB with different chitosan amounts.

MB Samples with Chitosan Concentration, %	pH_pzc_	-OH (phen.), w%	-NH_2_, w%	-COOH, w%	Total, w%
BSD	5.27 ± 0.06	2.2 ± 0.1	n.d. ^1^	1.7 ± 0.1	3.9 ± 0.1
MB-0%	4.37 ± 0.08	2.0 ± 0.1	n.d.	3.6 ± 0.1	5.6 ± 0.1
MB-2%	4.35 ± 0.08	1.5 ± 0.4	0.5 ± 0.4	2.9 ± 0.4	4.9 ± 0.7
MB-4%	4.42 ± 0.07	1.2 ± 0.1	0.6 ± 0.4	3.5 ± 0.1	5.2 ± 0.4
MB-10%	4.56 ± 0.17	1.3 ± 0.2	1.2 ± 0.2	3.0 ± 0.2	5.5 ± 0.3
MB-15%	4.57 ± 0.07	1.2 ± 0.1	1.6 ± 0.1	2.4 ± 0.2	5.2 ± 0.2

^1^ n.d.—not detected.

**Table 3:** Adsorption isotherm parameters for the Freundlich and Langmuir isotherm models for adsorption of Cu (II) and Cd (II) on the birch sanding dust (BSD) and chitosan mycelium bio-composites (MB) with different chitosan amounts (%) (pH 4.4–4.6 for Cu(II) and 4.7–4.9 for Cd(II), T = 22 °C).

MB Samples with Chitosan Concentration, %	1/n	n	K_F_	q_m_, mg g^−1^	K_L_, L/g
Cu (II) adsorption					
BSD	0.398	2.52	0.550	1.33	1.05
MB-0%	0.635	1.57	0.143	1.67	0.071
MB-2%	0.527	1.90	0.325	2.44	0.069
MB-4%	0.574	1.74	0.420	4.85	0.051
MB-10%	0.600	1.67	0.917	15.2	0.042
MB-15%	0.632	1.58	1.359	19.5	0.061
Cd (II) adsorption					
BSD	0.366	2.73	0.996	3.69	0.112
MB-0%	0.670	1.49	0.173	5.43	0.027
MB-2%	0.562	1.78	0.279	3.60	0.055
MB-4%	0.483	2.07	0.271	2.79	0.105
MB-10%	0.592	1.69	2.997	3.75	0.047
MB-15%	0.673	1.49	0.996	5.03	0.033

## Data Availability

The original contributions presented in this study are included in the article. Further inquiries can be directed to the corresponding author.

## Abbreviations

The following abbreviations are used in this manuscript:

BSD	Birch (*Betula pendula*) sanding dust
MB	Mycelium bio-composite
pH_pzc_	Point of zero charge
